# A New Technique for Surgical Treatment of Vaginal Agenesis Using Combined Abdominal-Perineal Approach

**DOI:** 10.1155/2011/120175

**Published:** 2011-04-26

**Authors:** Mehmet Sinan Beksac, Mehmet Coskun Salman, Nasuh Utku Dogan

**Affiliations:** Department of Obstetrics and Gynecology, Hacettepe University Faculty of Medicine, Sihhiye, Ankara 06100, Turkey

## Abstract

Optimum therapeutic approach in vaginal agenesis has always been an area of extensive controversies. Although surgical management gained priority due to the evolution of techniques, there is currently no consensus in the literature regarding the best type of surgical approach. The most commonly preferred surgical procedure among gynecologists is McIndoe operation which involves the creation of a space between bladder and rectum, insertion of a mold covered with split-thickness skin graft into that neovaginal space, and use of postoperative vaginal dilation to avoid stenosis. However, many modifications have been introduced in time in an attempt to increase the success rates. In this paper, we describe two cases with vaginal agenesis with functioning uterus who were subjected to surgery by combined abdominal-perineal approach. The surgical technique also included the use of a specially designed vaginal mold made up of polymethyl methacrylate and use of Hyalobarrier gel which is an adhesion-preventing agent.

## 1. Introduction

Vaginal agenesis is a rare condition with an incidence ranging from 1 in 4,000 to 1 in 10,000 females [1]. The most common etiology is Rokitansky-Küster-Hauser-Mayer (RKHM) syndrome or Mullerian agenesis which is described as congenital absence of uterus and vagina in an individual with normal female genotype, normal secondary sexual characteristics, and normal ovaries. Vaginal agenesis may also occur as an isolated abnormality [2, 3]. 

Detection of vaginal agenesis is readily achieved through well-defined diagnostic steps performed for primary amenorrhea at puberty [2]. However, the optimum therapeutic approach has always been an area of extensive controversies. Although surgical management gained priority due to the evolution of techniques, there is currently no consensus in the literature regarding the best type of surgical approach [4]. The most commonly preferred surgical procedure among gynecologists is McIndoe operation which involves the creation of a space between bladder and rectum, insertion of a mold covered with split-thickness skin graft into that neovaginal space, and use of postoperative vaginal dilation to avoid stenosis [3, 5, 6]. Many modifications have been introduced in course of time regarding the mold features, surgical technique, and postoperative care to obtain increased therapeutic success with relatively low morbidity. 

The aim of this paper is to report two cases of vaginal agenesis with functioning uterus who were subjected to a new surgical technique including the use of a specially designed vaginal mold made up of polymethyl methacrylate and use of Hyalobarrier gel, an agent originally used to prevent postoperative adhesions.

## 2. Case One

A 14-year-old girl who had normal female genotype and normal secondary sexual characteristics presented with primary amenorrhea and cyclic pelvic pain. Pelvic examination revealed vaginal dimple without a normal vaginal orifice, and an enlarged uterus was palpated on rectoabdominal examination. On pelvic magnetic resonance imaging (MRI), the uterus which was enlarged due to hematometra was *deviated to left side, and *a left-sided hematosalpinx was detected. The ovaries looked normal. Surgical treatment with a preoperative diagnosis of vaginal agenesis was scheduled after a detailed counseling regarding the surgical method and its possible complications. 

**Figure 1:**
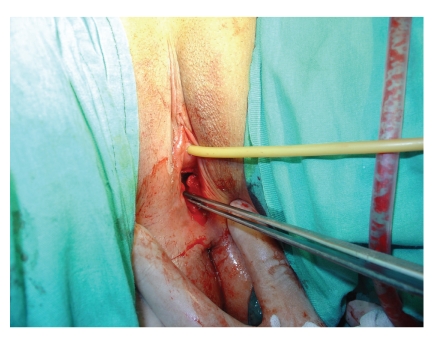
Neovaginal space created by blunt dissection.

The patient had an overnight fasting before the day of surgery. A single intravenous dose of 1000 mg cefazolin was given 30 minutes before the operation for antimicrobial prophylaxis. Under general anesthesia, the patient was placed in the dorsal lithotomy position. However, in order to allow the combined perineal-abdominal approach, the lower extremities placed in stirrups were positioned with the hips abducted 45 degrees and flexed 45 degrees. After catheterization of bladder, the abdominal and vulvar areas were cleaned and draped. The operation began with a transverse incision at the site of vaginal dimple. A potential space was created between the bladder and urethra in front and rectum behind by blunt dissection. The surgeon palpated the catheter in bladder and assistant's finger in rectum to avoid vesical or rectal injury during the dissection. The dissection continued until a neovagina was obtained with a length of 9-10 cm and a diameter of 3-4 cm. The walls of the cavity were carefully checked for hemostasis (Figure 1). 

**Figure 2:**
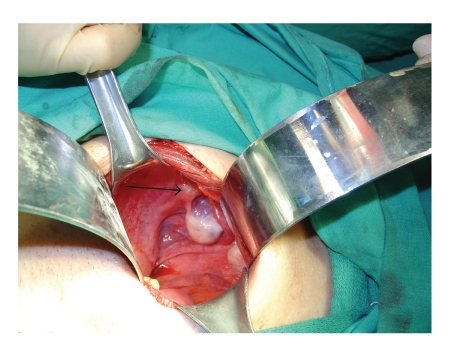
Right rudimentary uterine horn (black arrow) with normal tube and ovary.

**Figure 3:**
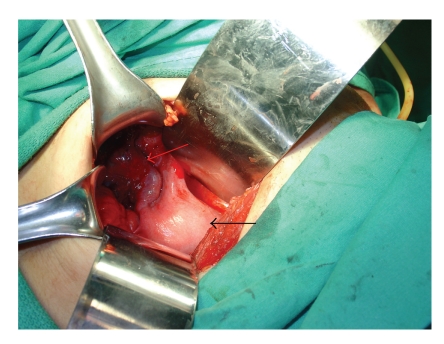
Enlarged left uterine horn (black arrow) with hematosalpinx (red arrow).

**Figure 4:**
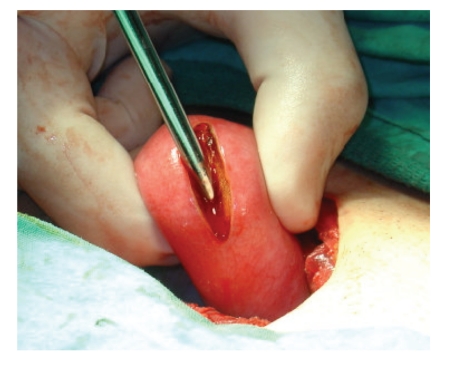
Uterine cavity catheterized with a hysterometer which is pushed downward until neovaginal space was accessed.

**Figure 5:**
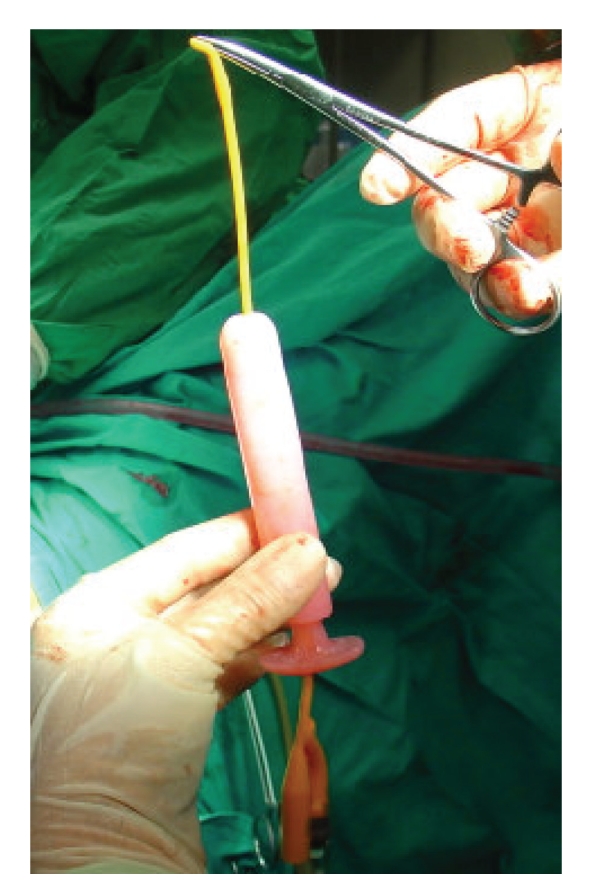
The specially designed vaginal mold with an 8-french pediatric Foley catheter placed in its central lumen.

**Figure 6:**
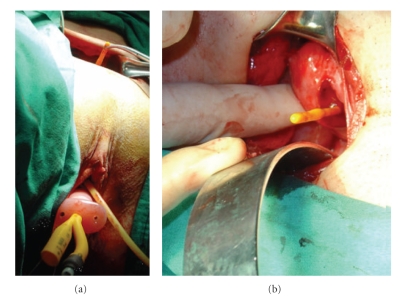
The Foley catheter passing through the lumen of the mold with its tip located in the uterine cavity ((a) and (b)).

**Figure 7:**
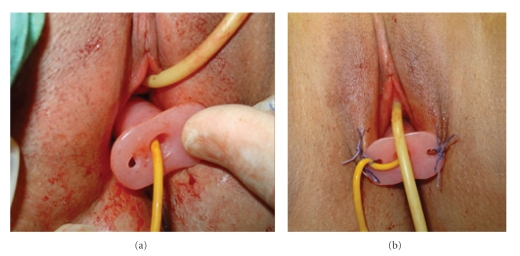
The mold in neovagina (a) and sutures put in between labia majora and the small holes located at the distal end of the mold (b).

After the creation of neovagina, a second surgeon performed a laparotomy via pfannenstiel incision. On exploration, both ovaries and right Fallopian tube were normal. A rudimentary uterine horn was detected on the right side which seemed to be nonfunctioning, and no hematometra or hematosalpinx was evident (Figure 2). On the left side, a relatively enlarged uterine horn and hematosalpinx were seen (Figure 3). Hematoma within the left tube was drained, and neosalpingostomy was performed which was secured via eversion provided by 3.0 polyglactin sutures. A hysterotomy was performed subsequently on right anterolateral fundal portion of left uterine horn. The uterine cavity was identified, and hematometra was drained. The cavity was catheterized with a hysterometer which was pushed slowly downward until neovaginal space was accessed (Figure 4). The hysterometer used was a straight, graduated, and sounding metal hysterometer with a rounded tip which was slightly conical with outside diameters ranging from 3 to 5 millimeters. The specially designed vaginal mold was inserted into the neovagina with an 8-french pediatric Foley catheter placed in its central lumen (Figure 5). The tip of the Foley catheter was located in the uterine cavity, and its balloon was insufflated with 3 mL saline (Figure 6). Endometrium and myometrium were closed separately with 2.0 interrupted polyglactin sutures. To maintain the mold in position, sutures were put in between labia majora and the small holes located at the distal end of the mold with 1.0 polyglactin sutures (Figure 7). Hyalobarrier gel was applied between neovaginal walls and the mold. 

The bladder catheter and the mold were maintained for 10 days postoperatively. Because mobilization was restricted, low-molecular-weight heparin was administered twice a day, and the patient was given low-residue diet during this period. After removing the mold, the patient was advised to use the mold almost constantly for 3 months postoperatively and at nights for the subsequent 3 months to prevent stricture of the neovagina. The mold had to be cleaned on a daily basis, and the patient was explained the method of cleaning and inserting the mold on her own. 10% iodine and 3% hydrogen peroxide solutions were used, respectively, for cleaning the mold. Then, for lubrication and to support an enhanced epithelization, the mold was covered with 0.1% estriol cream just before the insertion. Gauze bandages tied around the waist and upper calves were used to maintain the mold in the neovagina after removing the sutures put in between labia majora and the mold. The patient was discharged from hospital on 14th postoperative day with an uneventful postoperative course. Her last visit was 1 year after the operation, and she experienced regular menses without pain. Her neovagina was patent on examination with normal cervix and uterus.

## 3. Case Two

A 16-year-old girl having normal female genotype and normal secondary sexual characteristics presented with primary amenorrhea and cyclic pelvic pain. On pelvic examination, she did not have vaginal orifice, and her uterus was enlarged on rectal examination. Pelvic MRI could not detect a normal vagina, but uterus was filled with blood and proximal vagina was dilated due to proximal hematocolpos. The ovaries were normal. After counseling the patient and her family, surgery was planned with a preoperative diagnosis of distal vaginal agenesis. 

After similar preoperative preparation and anesthesia induction, bladder was catheterized, and abdominal and vulvar areas were cleaned and draped. A transverse incision was done on vaginal dimple. A space with a length of 4-5 cm and a diameter of 2-3 cm was created by blunt dissection until proximal vagina was reached and hematometra and proximal hematocolpos drained. Hemostasis was achieved.

Second surgeon started laparotomy via pfannenstiel incision. On exploration, ovaries, tubes, and uterus were normal. Hysterotomy was performed on uterine fundus to identify endometrial cavity. The cavity was catheterized with a hysterometer which extended into neovaginal space. Vaginal mold was inserted into the neovagina with Foley catheter placed in its central lumen in a similar fashion to that already described for the former patient. Balloon of Foley was insufflated and left in the uterine cavity. Endometrium and myometrium were closed, the mold was fixed to labia majora, and Hyalobarrier gel was applied on neovaginal walls.

Postoperative followup was similar, and the same protocol was used. The patient was discharged from hospital on 15th postoperative day. Her last examination was 16 months after the surgery when she reported regular and painless menses and her examination revealed normal vaginal cavity, cervix, and uterus.

## 4. Discussion

Vaginal agenesis is an uncommon condition which is encountered for a few times during the professional career of a general gynecologist [1, 3]. However, the diagnosis may considerably unsettle the girl or the young adolescent and her family. Therefore, attention must be given to the psychosocial issues as well as to the correction of the anatomical abnormality [7]. Also, counseling about future fertility is of vital importance. However, normal future fertility may be expected in patients with isolated vaginal agenesis in whom normal functioning uterus and intact cervix are present. On the other hand, future fertility problems may be encountered due to hematosalpinx, endometriosis, and the surgery itself which should be emphasized during counseling. 

The timing for both nonsurgical and surgical correction of this anomaly is purely elective [7]. Nevertheless, in patients with intact uterus experiencing cyclic pelvic pain due to the obstructed menstrual fluid flow, immediate surgical correction is mandatory at the time of diagnosis. In the literature, although more than 10 surgical procedures for neovaginal reconstruction have been described so far, an ideal approach has not yet been identified [8, 9]. Especially vaginal molds which are used to prevent restenosis of created neovagina may be associated with most of the problems. Poor drainage, graft maceration, sloughing, and graft detachment may be caused by nonideal vaginal molds and lead to an unsatisfactory reconstruction. Using an appropriate vaginal mold is one of the keys to achieving a successful result in McIndoe neovaginal reconstruction [10, 11]. Since the first operation had had the best chance of success, we were motivated to seek a new vaginal mold that has advantages for clinical use. For this purpose, we designed special mold which may specifically be used for pubertal girls with complete or partial vaginal agenesis and a normal uterus where a patent uterocervicovaginal canal is required to allow normal flow of menstrual and mucosal fluids. The mold is made up of polymethyl methacrylate which is commonly used for the production of most denture base prosthetics. Therefore, it is a relatively simple and a cheap mold which may be readily produced in dentistry laboratories in a short period of time. The mold has a length of 9-10 cm and a diameter of 2-3 cm. It has a central lumen in which an 8-french pediatric Foley catheter is placed. The tip of the Foley with the insufflated balloon is placed and left in the uterine cavity to allow the drainage of blood during the early postoperative period. Also, the Foley allows irrigation with saline which may be needed to facilitate the drainage of bloody fluid or to manage postoperative local infection. The mold we designed has small holes located at both sides of the distal end which is used to put sutures in between labia majora and the mold to maintain the mold in position and to prevent its displacement during the postoperative period. 

The surgical technique we described has not only involved the use of a specially designed vaginal mold, but also Hyalobarrier gel was used which was applied between neovaginal walls and the mold at the end of the surgery. Hyalobarrier is an autocrosslinked derivative of hyaluronan (hyaluronic acid, HA) which is a natural polysaccharide found in almost all tissues and body fluids. HA plays a role in maintaining the structural integrity of tissues. Hyaluronan has been recognized in clinical medicine due to its high degree of biocompatibility, favorable safety profile, tissue protective properties, and positive biological effects on healing. The autocrosslinking process increases viscosity and extends in vivo residence time while preserving the favorable properties of hyaluronan. Also, the only product of in vivo degradation is natural HA since no foreign molecules are used during this process [12, 13]. During the healing process, prolonged presence of HA in increased amounts was shown to be associated with lack of fibrosis and scar formation [14]. Also, exogenous HA was reported to decrease the synthesis of collagen in human dermal fibroblasts without effecting endogenous HA synthesis [15]. Actually, deposition of hyaluronan around surgically treated tissues reduces postoperative adhesion formation [16]. In this context, use of Hyalobarrier gel was associated with a reduction in the incidence and severity of postoperative adhesions after hysteroscopy, laparoscopy, and laparotomy [17, 18]. Highly viscous Hyalobarrier gel provides a biodegradable physical barrier that limits the contact among injured sites for a protracted period [19]. Hyalobarrier gel use was considered in this patient due to the assumption that presence of high amounts of HA on the surgical wound may cause a healing process without fibrosis and scarring. Given the stenosis of vagina mostly caused by the thick scar just at the surgical site, a second failure might be observed with surgery only. In fact, successful use of Hyalobarrier gel in such a condition was first reported by our team in the literature previously [20]. As a result, use of adhesion-preventing agents may be considered in women who are scheduled for the surgical correction of vaginal agenesis. 

Finally, when a patient is diagnosed to have vaginal agenesis, she and her family should be counseled in detail before the surgery. The surgical method with its possible complications including bladder and rectum injury should be discussed. Also, they should be informed about relatively long postoperative care period of almost 6 months which is critical for the success of the surgery when vaginal mold is removed and inserted on a daily basis. If postoperative recommendations are not performed properly, neovaginal space may be closed, and reobstruction of menstrual flow may occur which necessitates further surgical interventions. 

In conclusion, in female patients with complete or partial vaginal agenesis and normal, functioning uterus, a combined perineal-abdominal surgical approach may be performed successfully in association with the use of specially designed vaginal mold and Hyalobarrier gel. However, further studies are needed to warrant the use of this surgical technique of combined perineal-abdominal approach in association with this type of mold and Hyalobarrier gel or other adhesion-preventing agents in patients with vaginal agenesis. Also, abdominal component of the surgery can be completed laparoscopically in experienced hands which may decrease the morbidity and provide a faster postoperative recovery compared to laparotomy.
